# Pott’s Puffy Tumor in the Adult Population: Systematic Review and Meta-Analysis of Case Reports

**DOI:** 10.3390/jcm14124062

**Published:** 2025-06-08

**Authors:** Klaudia Kokot, Justyna Małgorzata Fercho, Konrad Duszyński, Weronika Jagieło, Jakub Miller, Oskar Gerald Chasles, Rami Yuser, Martyna Klecha, Rafał Matuszczak, Eryk Nowiński, Kaja Klein-Awerjanow, Tomasz Nowicki, Maciej Mielczarek, Jacek Szypenbejl, Mariusz Siemiński, Tomasz Szmuda

**Affiliations:** 1 Scientific Circle of Neurotraumatology, Department of Emergency Medicine, Medical University of Gdańsk, 80-210 Gdańsk, Poland; kduszynski@gumed.edu.pl (K.D.); weronikajagielo@gumed.edu.pl (W.J.); oskar.chasles@gumed.edu.pl (O.G.C.); ramiyuser@gumed.edu.pl (R.Y.); 2 Emergency Department, Medical University of Gdańsk, University Clinical Centre in Gdańsk, 80-952 Gdańsk, Poland; jacek.szypenbejl@gumed.edu.pl (J.S.); mariusz.sieminski@gumed.edu.pl (M.S.); 3 Neurosurgery Department, Swissmed Luxmed Hospital in Gdańsk, 80-215 Gdańsk, Poland; tomasz.szmuda@gumed.edu.pl; 4 Neurosurgery Department, 10th Military Clinical Hospital with PolyClinic SPZOZ in Bydgoszcz, 85-681 Bydgoszcz, Poland; 5 Graduate School of Informatics, University of Amsterdam, 1012 WX Amsterdam, The Netherlands; jakubmiller@gmail.com; 6 Otolaryngology Department, Medical University of Gdańsk, University Clinical Centre in Gdańsk, 80-952 Gdańsk, Poland; martyna.klecha@gumed.edu.pl (M.K.); raf.matuszczak@gumed.edu.pl (R.M.); 7 Anaesthesiology Department, Medical University of Gdańsk, University Clinical Centre in Gdańsk, 80-952 Gdańsk, Poland; nowinskieryk71@gmail.com; 8 Radiology Department, Medical University of Gdańsk, University Clinical Centre in Gdańsk, 80-952 Gdańsk, Poland; kaja.klein-awerjanow@gumed.edu.pl (K.K.-A.); tnowicki@gumed.edu.com.pl (T.N.); 9 Neurosurgery Department, Stanisław Staszic Specialist Hospital, 64-920 Piła, Poland; maciejmielczarek87@gmail.com; 10 Neurosurgery Department, Medical University of Gdańsk, University Clinical Centre in Gdańsk, 80-952 Gdańsk, Poland

**Keywords:** Pott’s puffy tumor, frontal osteomyelitis, subperiosteal abscess, sinusitis complication, frontal sinusitis complication

## Abstract

**Objectives**: Pott’s puffy tumor (PPT) is a rare and life-threatening infection of the frontal sinuses, predominantly affecting children but with less frequent reports in adults. Therefore, we present an analysis of one hundred and eighty-one cases of adult patients diagnosed with PPT, along with a description of one of our cases. The purpose of this research is to identify the most common symptoms, predisposing medical history, predominant microorganisms, commonly used antibiotics, treatment options, long-term outcomes, and possible complications in adults. Despite its rarity, PPT has a dynamic course, necessitating familiarization with appropriate treatment methods to improve patient well-being. **Methods**: Methods involved a systematic search of PubMed, Medline, Google Scholar, Web of Science, EBSCO, and Scopus, following PRISMA guidelines. A total of 122 articles were screened, providing 180 adult patients aged 18 to 86, alongside 1 additional patient treated at our institution, bringing the total to 181 patients. **Results**: The results showed that the patients ranged from 18 to 86 years of age (mean age of 47 years), with 72.2% being males. The most common symptoms were forehead swelling (74.7%), frontal headache (67%), fever (59.3%), and acute/chronic rhinosinusitis (39.6%). The risk factors associated with its development include sinusitis (49.5%) and previous head trauma (12.6%). Intracranial involvement was found in 38.1% of patients. Streptococcus spp. (19.3%) and Staphylococcus spp. (16.6%) were the most commonly identified pathogens. Surgical intervention was employed in 87.3% of cases, with a mean hospital stay of 23 days. There was no significant difference in hospital stay or rehospitalization rates between those with and without intracranial involvement. Antibiotic therapy was used in 87.3% of cases, with a mean duration of 61 days. A combination of Cephalosporin, Metronidazole, and Nafcillin was the most common empirical antibiotic therapy. The mean follow-up period was 14 months, with a mortality rate of 1.6%. **Conclusions**: The conclusion highlights the importance of the prompt initiation of empirical antibiotic therapy, followed by targeted treatment based on microbiological cultures. Recognizing that PPT symptoms are not exclusive to pediatric patients but can also affect adults is crucial. PPT warrants further research to optimize its management and outcomes. It is believed that PPT may be more treatable in adults when identified early, which emphasizes the need for PPT recognition among adults. Timely empirical antibiotics based on microbiological results, along with appropriate surgical intervention, are critical for improving outcomes. Multidisciplinary care involving otolaryngologists, neurologists, and infectious disease specialists is essential. Further studies should be developed for the evaluation of diagnostic protocols and long-term management strategies.

## 1. Introduction

Pott’s puffy tumor (PPT) is a rare, potentially life-threatening medical condition characterized by osteomyelitis of the frontal bone with subperiosteal abscess formation. Since its initial recognition in the late 1760s by English surgeon Sir Percivall Pott, only a few hundred cases have been reported, with most records emerging in the last decade. Although PPT can occur across all age groups, it is more frequently observed in adolescents than in younger children or adults. This is thought to be due to the more advanced pneumatization of the frontal sinuses during adolescence, which may facilitate the spread of infection to the frontal bone [1]. We present the largest meta-analysis in the literature, encompassing 181 adult cases of PPT, including an illustrative description of our own case. Etiological factors predisposing individuals to PPT include the extension of infection from the frontal sinus, trauma to the frontal bone, substance use (especially intranasal use), odontogenic disease, and mastoiditis. Despite its rarity, PPT often arises as an uncommon but serious complication of sinusitis—a condition that affects millions worldwide each year [2].

Acute rhinosinusitis (ARS) has a one-year prevalence of 6–15% and is usually a consequence of a viral common cold. Chronic rhinosinusitis (CRS), on the other hand, is a significant health problem affecting 5–12% of the general population [3]. ARS and CRS are generally mild diseases, but 0.5 to 2% of patients develop acute bacterial rhinosinusitis. Most cases resolve without consequences. However, in prolonged or severe cases, serious complications such as PPT can occur, sometimes with intracranial involvement.

PPT cases described in the literature are predominantly in the form of case reports or short literature reviews. Larger meta-analyses concern either pediatric populations or both pediatric and adult populations [2,4]. The aim of this study is to comprehensively present symptoms, diagnostics, microbiological considerations, surgical and pharmacological treatment methods, and prognosis solely in adult patients.

## 2. Case Presentation

A 40-year-old man was admitted to the Emergency Department due to a 3 cm, non-tender swelling in the frontal region that appeared a day earlier. He had been suffering from a severe headache for one week and pain in the left ear for 4 days. Although his neurological examination was intact, the patient was hyperactive, ignoring requests from the medical team. He had been seen 2 days earlier by a general practitioner, who prescribed an unknown oral antibiotic.

**Figure 1:**
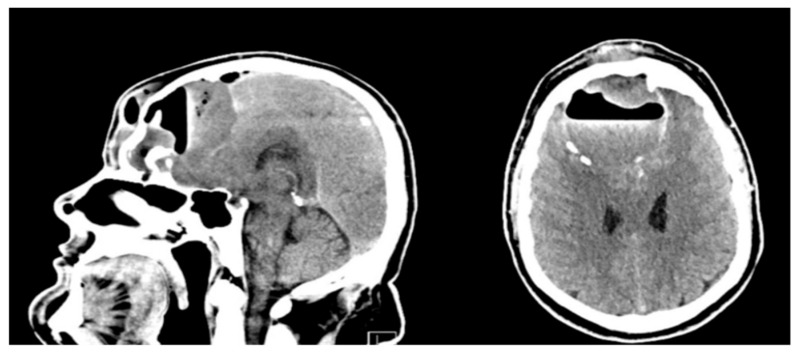
Head CT of the patient before surgery, showing a large structure with an air-fluid level, small gas bubbles, and peripheral calcifications bilaterally in the frontal lobes, brain tissue edema, a 4 mm midline shift, and subfalcine herniations, accompanied by destruction of the posterior frontal lobe. The imaging highly suggested PPT.

An initial empiric antibiotic therapy was administered and consisted of third-generation Cephalosporin and Metronidazole, which is consistent with the literature. The patient underwent ultrasound of the frontal sinus and computed tomography (CT) of the head (Figure 1). The head CT demonstrated a large structure with an air-fluid level, small gas bubbles, and peripheral calcifications bilaterally in the frontal lobes, brain tissue edema, a 4 mm midline shift, and subfalcine herniations.

The patient was diagnosed with PPT and underwent surgery in a planned accelerated mode. During the craniotomy, the partially calcified dura mater was excised. Cranialization of the frontal sinuses, implantation of a drain under the bone flap, and a graft into the frontal sinus outlet with a fragment of the temporal muscle were performed. Pseudomonas aeruginosa was cultured from the collected samples. Antibiotic therapy was adjusted to the administration of Sulperazon (Cefoperazone + Sulbactam) at a dose of 2 × 2 g i.v. for 14 days.

**Figure 2:**
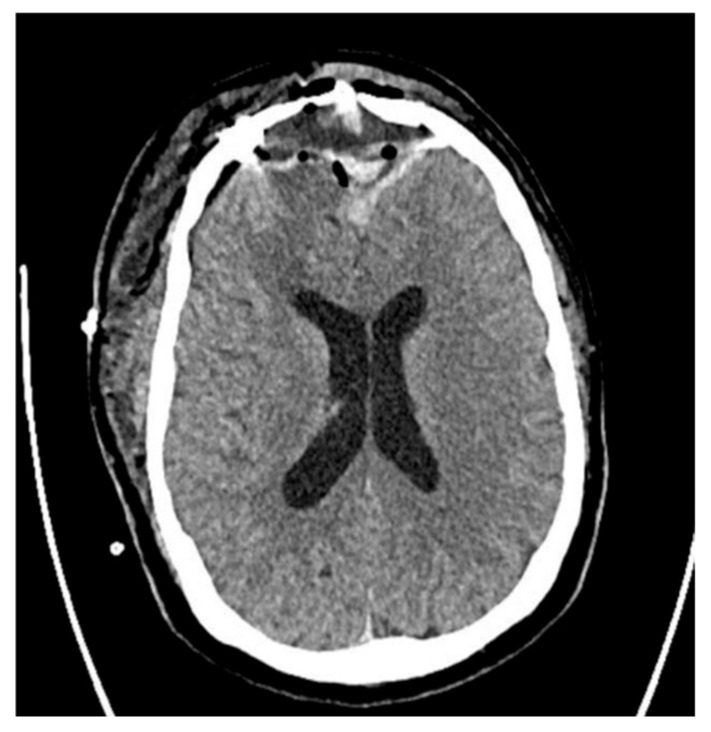
Postoperative head CT showing resolution of brain edema, implanted drains, and the bone graft.

Postoperative CT showed significant regression of the lesions (Figure 2). After 18 days of hospitalization, the patient was discharged in a generally good condition, and he has remained under the care of the neurosurgical outpatient department since, without any recurrence.

## 3. Materials and Methods

A systematic literature review was conducted in accordance with the PRISMA 2020 guidelines. A comprehensive search of the PubMed, Medline, Google Scholar, Web of Science, EBSCO, and Scopus databases was carried out from inception to January 2024. The search terms included the following: “Pott’s puffy tumour”, “frontal osteomyelitis”, “subperiosteal abscess”, “sinusitis complication”, and “frontal sinusitis complication”. Articles were filtered by age (≥18 years old) and language (English, Polish, Spanish, Chinese or German). The following inclusion criteria were applied:

- Patients with a radiologically or surgically confirmed diagnosis of PPT;
- Patients aged 18 years and older;
- Complete case description, including symptoms, imaging, treatment, and outcome.

The exclusion criteria were

- Pediatric patients (<18 years old);
- Unavailable full-text articles;
- Articles published in languages other than English, Polish, Spanish, Chinese, or German;
- Articles that were not peer-reviewed.

In addition to the systematic search, the reference lists of included studies were reviewed for any relevant missed publications. All relevant data, including patient demographics, presenting symptoms, imaging findings, microbiological cultures, treatment modalities, and outcomes, were extracted and compiled into a structured database. Descriptive statistics were used to analyze the data, and the results are presented in the form of frequencies and percentages. The date of publication and country of origin were included in the analysis. All statistical tests and graphical illustrations were conducted using Looker Studio, Google Sheets (Google LLC, Mountain View, CA, USA), and GraphPad Prism version 10.4.1. The review was not registered in PROSPERO.

## 4. Results

### 4.1. Description of the Included Studies

**Figure 3:**
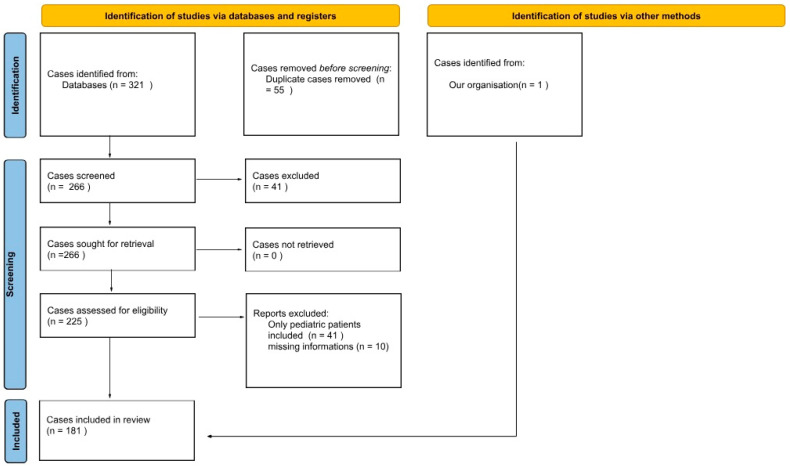
PRISMA protocol of cases.

**Figure 4:**
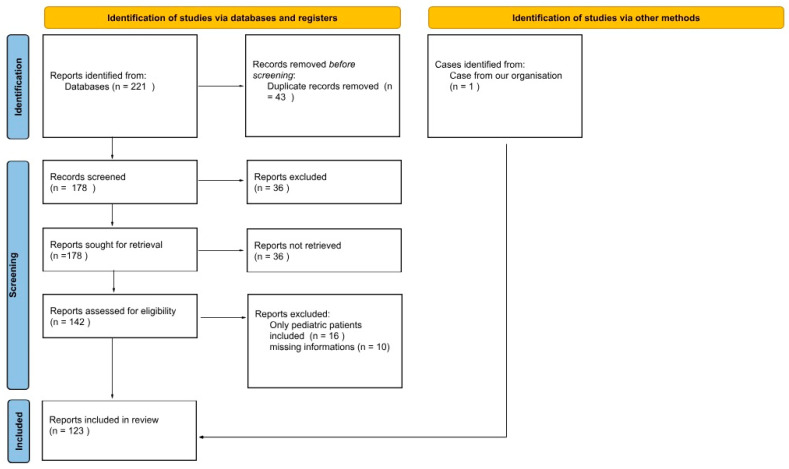
PRISMA protocol of articles.

We included 123 articles, in which 180 adult patients with PPT were described. An additional case from our hospital records was also included, yielding a total of 181 patients (Figure 3). Detailed clinical data are presented in Supplementary Table S1. The initial strategy generated 221 articles from all databases. After duplicate removal, 178 articles underwent an independent full-text review by three investigators, and 123 articles were included for the final review (Figure 4). We added our own case and another 6 cases searched for by hand in databases from the resources of the Medical University of Gdańsk’s library. Articles were predominantly case series or case reports [1,5,6,7,8,9,10,11,12,13,14,15,16,17,18,19,20,21,22,23,24,25,26,27,28,29,30,31,32,33,34,35,36,37,38,39,40,41,42,43,44,45,46,47,48,49,50,51,52,53,54,55,56,57,58,59,60,61,62,63,64,65,66,67,68,69,70,71,72,73,74,75,76,77,78,79,80,81,82,83,84,85,86,87,88,89,90,91,92,93,94,95,96,97,98,99,100,101,102,103,104,105,106,107,108,109,110,111,112,113,114,115,116,117,118,119,120,121,122,123].

**Figure 5:**
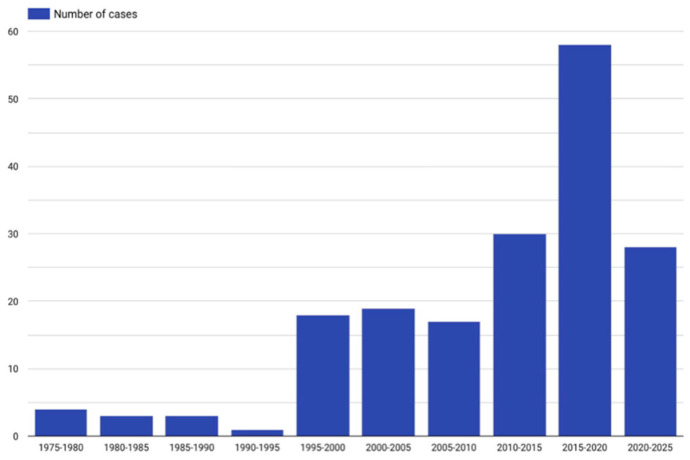
Number of published cases in specific years.

Patients ranged from 18 to 86 years in age (average of 46.1 years, median 47 years). In our study, 72.4% (n = 131) were male and 27% (n = 49) were females, and for 0.6% (n = 1), sex was not mentioned. One woman was pregnant. In addition, 20.4% (n = 37) were White, 3.3% (n = 6) were African, 8.3% (n = 15) were Asian, 0.6% (n = 1) were Native Americans, 0.6% (n = 1) were Saudi Arabian, 0.6% (n = 1) were Native Australian, and for 66.3% (n = 120), their race/nationality was unknown. Figure 5 presents the specific number of published articles on PPT.

### 4.2. Results of the Meta-Analysis

**Figure 6:**
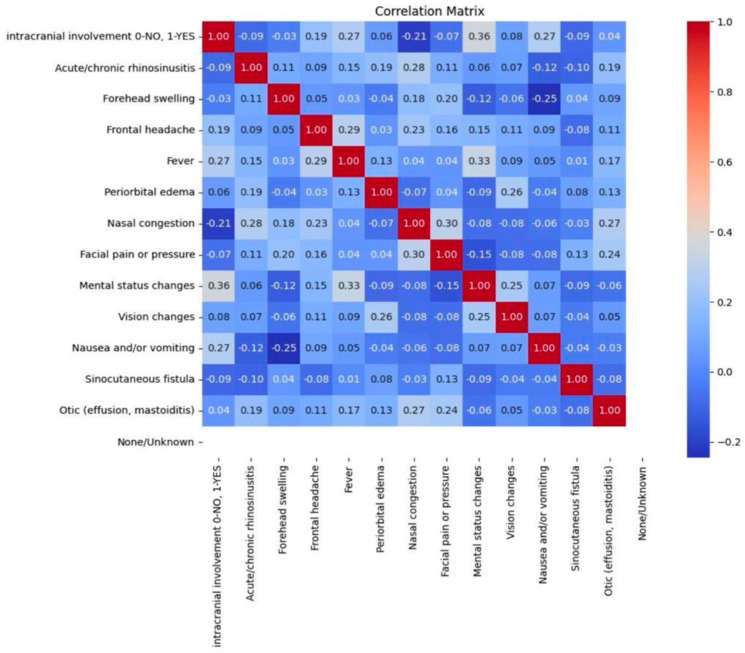
Correlation matrix of clinical signs and symptoms presented in PPT patients, illustrating statistical relationships between them. Strong associations demonstrate patterns of co-occurring symptoms. The greatest statistical associations were identified between nausea and/or vomiting and forehead swelling (−0.25, *p* < 0.001), intracranial involvement and mental status changes (0.36, *p* < 0.0001), and fever and mental status changes (0.33, *p* < 0.0001).

**Figure 7:**
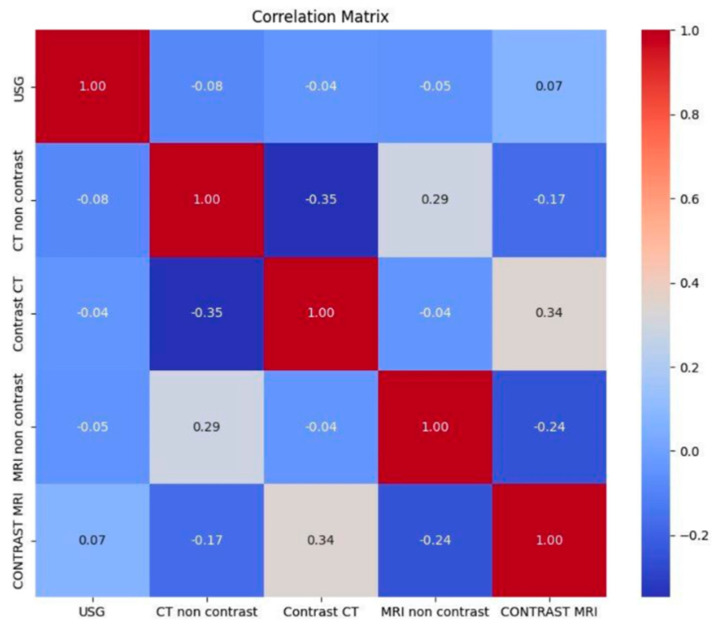
Correlation matrix of imaging procedures used during the diagnostic process of PPT. Extended diagnostics for non-enhanced magnetic resonance imaging (NE-MRI) after non-enhanced CT (NE-CT) was significant.

A total of 20 causes of PPT are reported in the literature. Among these, 14 are symptoms of diseases that may lead to PPT and 5 are radiological imaging features that have been identified as statistically significant predictors of PPT occurrence (Figure 6 and Figure 7).

### 4.3. Subgroup of Meta-Analysis

#### 4.3.1. Signs and Symptoms

**Figure 8:**
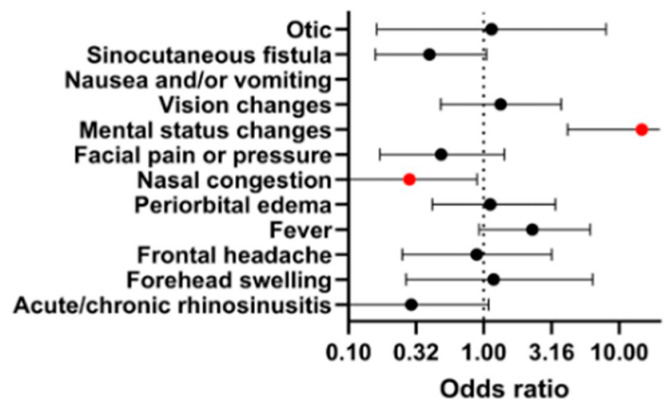
Forest plot of reported PPT symptoms and intracranial involvement. An odds ratio below 1 indicates a higher prevalence of symptoms in tumors without intracranial involvement, and an odds ratio above 1 indicates higher prevalence in tumors with intracranial involvement. Statistically significant (*p* < 0.05) data points are marked in red. The odds ratio for nausea and/or vomiting is positive infinity (*p* < 0.01).

The most frequently reported symptoms of PPT were forehead swelling (74.7%), frontal headache (67%), fever (59.3%), acute/chronic rhinosinusitis (39.6%), periorbital edema (33.5%), facial pain or pressure (28.6%), nasal congestion (24.2%), sinocutaneous fistula (20.3%), mental status changes (14.3%), vision changes (13.7%), nausea and/or vomiting (4.4%), and otitis effusion or mastoiditis (2.2%). The odds ratio of intracranial involvement and the occurrence of given symptoms is presented in Figure 8. The presence of nasal congestion exhibited statistical significance (*p* < 0.05) in association with intracranial involvement. Meanwhile, nausea and/or vomiting and mental status changes were statistically significant (*p* < 0.01 and *p* < 0.0001, respectively) for tumors without intracranial involvement.

#### 4.3.2. Medical History

The factors most frequently associated with PPT in our cohort were sinusitis (49.5%), prior head trauma (12.6%), diabetes (9.3%), smoking (4.9%), odontogenic disease (4.9%), rheumatologic disorders (3.3%), history of substance use (2.2%), and obesity (1.6%). It is important to emphasize that these factors represent associations observed in the studied population, and causality cannot be definitively established based on the available data.

#### 4.3.3. Radiological Imaging

The most frequent imaging modalities were non-enhanced CT (NE-CT) (48.4%), non-enhanced MRI (NE-MRI) (24.7%), contrast-enhanced CT (CE-CT) (23.1%), and contrast-enhanced MRI (CE-MRI) (15.4%). The least common examination was ultrasound (3.8%, n = 7). In 24.7% of patients, no imaging was performed—this may reflect limited access to imaging in earlier years. Furthermore, 22.5% of patients who had NE-CT underwent further diagnostic evaluation with MRI.

#### 4.3.4. Microbiology and Laboratory Workup

**Figure 9:**
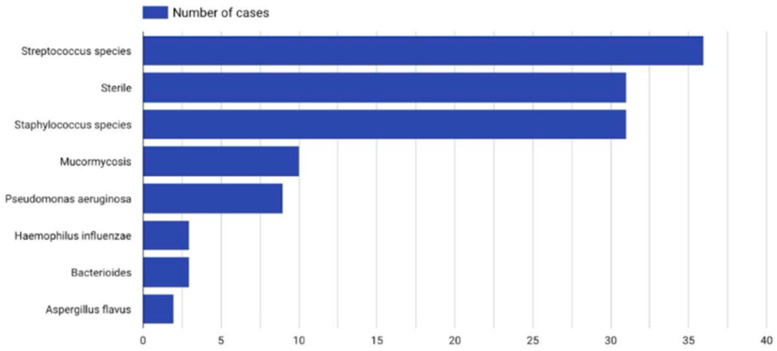
Distribution of bacterial species in cultured samples.

From the collected material, it was found that pathogens were cultured in 51.9% of patients. In 21.5%, no bacteria were cultured, and in 26.5%, data were not available. The most common pathogens were Streptococcus species (19.3%), Staphylococcus species (16.6%), Pseudomonas aeruginosa (6%), and Mucormycosis (5.5%). Less frequent pathogens are presented in Figure 9.

#### 4.3.5. Intracranial Involvement

Intracranial involvement was found in 38.1% of cases, with a mean age of 40 years in this group. Antibiotic therapy was used in 98.6% of patients with and 80% of patients without intracranial involvement. The duration of oral antibiotic therapy did not differ significantly between those with and without intracranial involvement. However, the duration of intravenous therapy was almost twice as long in patients without intracranial involvement (61 vs. 37 days, respectively). Notably, the presence of intracranial involvement did not significantly affect the length of hospitalization or the rate of rehospitalization.

#### 4.3.6. Treatment Method

Overall, 87.3% of patients underwent surgical treatment. The most common methods included a combined approach (57 cases), external drainage (54 cases), and endoscopic sinus surgery (ESS) (41 cases). Ten patients did not undergo surgery. In 14 cases, the treatment method was not mentioned. The average follow-up in surgically treated patients was 15 months, whereas in non-surgical patients, it was only 3 months. The duration of antibiotic therapy was almost two times shorter in patients who underwent surgical treatment.

#### 4.3.7. Antibiotic Therapy

**Figure 10:**
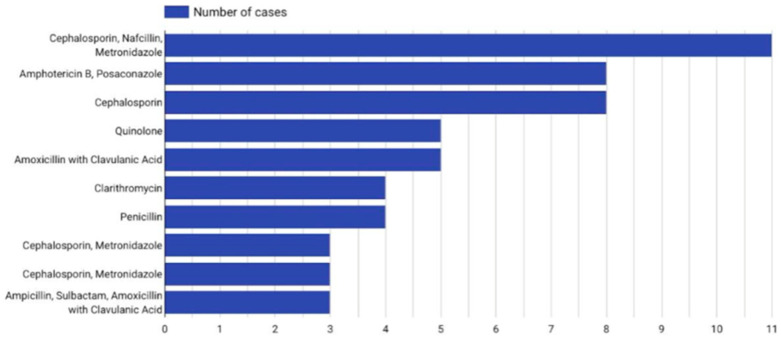
Histogram of most common therapeutic combinations—distribution of antibiotic schemes by frequency of use.

Antibiotic therapy was used in 87.3% of patients. The mean duration of intravenous therapy was 51 days, that of oral therapy was 33 days, and that of any antibiotic therapy was 61 days. No significant difference in oral antibiotic duration was observed between patients with or without intracranial involvement (*p* = 0.203). Antibiotic polytherapy and antifungal treatment were used in 69.6% of cases. The most commonly used antibiotics were Cephalosporins (both in monotherapy and combinations), often combined with Metronidazole and Nafcillin. Therapy combinations are illustrated in Figure 10.

#### 4.3.8. Days of Hospitalization and Follow-Up

The mean duration of hospitalization was 23 days. Overall, 80% of the patients were rehospitalized, although no data regarding reasons was provided. The mean follow-up period was 14 months. The mortality rate was 1.6%.

## 5. Discussion

### 5.1. Epidemiology

According to the literature, Pott’s puffy tumor (PPT) is more commonly observed in the pediatric population. However, an increasing number of adult cases has been reported in recent years. This trend may be attributed both to a genuine rise in incidence and to improved detection resulting from the growing use of radiological imaging. Nevertheless, awareness of PPT and its associated symptoms remains limited and subject to debate. Based on our collected series, no correlation was found between the patients’ race and the presence of specific clinical symptoms associated with PPT.

### 5.2. Pathogenesis

The pathogenesis of PPT primarily involves two mechanisms: direct extension and hematogenous spread [5]. The most direct route often follows open trauma to the frontal region, which exposes the frontal bone to pathogens from the external environment [123]. In the majority of cases, PPT arises as a complication of bacterial sinusitis, with the infection spreading locally to involve the frontal bone.

The hematogenous route is more complex. Chronic bacterial overgrowth within the frontal sinus and surrounding soft tissues can disrupt the coagulation cascade in small blood vessels, leading to thrombosis and venous congestion. This impairs the perfusion of the frontal periosteum [5], triggering an inflammatory response that raises intracranial pressure and induces necrosis of the trabecular bone. These changes create a hypoxic, anaerobic environment conducive to the proliferation of opportunistic pathogens, ultimately resulting in abscess formation [5].

### 5.3. Signs and Symptoms

PPT symptoms usually emerge over several days to weeks, with a more acute course observed in children compared to adults [123]. Although rare, there are documented cases of patients experiencing intermittent forehead swelling over months or even years. In our study, the most common and rapidly developing symptoms were forehead swelling and frontal headache. Early signs such as facial pain, nasal congestion, and nasal discharge generally appear 4–5 weeks before medical consultation. Ocular symptoms—periorbital swelling and vision disturbances—typically manifest 1.5–2 weeks before diagnosis. A soft, well-demarcated forehead mass, nearly pathognomonic for PPT, usually develops within 1–2 weeks. Symptoms of increased intracranial pressure—nausea, vomiting, photophobia, seizures, mental status changes, and focal neurological deficits—tend to occur within the final week prior to diagnosis [2]. Interestingly, these symptoms were not among those most commonly reported in our dataset. PPT in the pediatric population is associated with a higher rate of intracranial involvement, whereas such complications are less common in adults [2].

### 5.4. Medical History

Gender was not found to influence the clinical presentation of symptoms. Consistent with the findings of Sandoval et al. [123], our study identified sinonasal infections and head trauma as the most common predisposing factors for Pott’s puffy tumor. Additionally, obesity and smoking emerged as significant contributing factors within the adult population. Notably, smoking has not been recognized as a risk factor in pediatric-focused studies, suggesting a potential shift in the risk profile when considering adult patients exclusively.

### 5.5. Radiological Imaging

According to Sharma et al. [124], when intracranial involvement is suspected, contrast-enhanced CT (CE-CT) should be the initial diagnostic modality. Intracranial complications, with or without frontal bone erosion, were present in 60–85% of such cases. CT is effective for detecting sinusitis, bone destruction, subperiosteal abscesses, and intracranial spread. However, MRI is considered superior for visualizing intracranial structures, particularly in identifying brain abscesses, hemorrhage, dural involvement, venous sinus thrombosis, and bone marrow edema. Diffusion-weighted imaging (DWI) is especially useful, as abscesses demonstrate restricted diffusion due to the presence of viscous pus. MRI also plays a crucial role in treatment monitoring and helps reduce radiation exposure [2]. Yet, due to the limited availability of MRI in many centers, non-enhanced CT remains the most accessible and timely diagnostic tool when PPT is suspected. Postoperative imaging using CT demonstrated marked resolution of the previously observed lesions (Figure 2). Following an 18-day inpatient course, the patient was discharged in stable condition, and he continues to be monitored through regular follow-ups at the neurosurgical outpatient clinic. No signs of recurrence have been observed to date. In alignment with evidence-based recommendations, prolonged antibiotic therapy following hospital discharge is essential to ensure complete bacterial clearance and to minimize the risk of relapse. Furthermore, the implementation of extended imaging protocols—particularly those incorporating contrast-enhanced CT and MRI—is widely advocated for in the literature as part of the comprehensive diagnostic and follow-up strategy in cases of Pott’s puffy tumor.

### 5.6. Microbiology and Laboratory Workup

PPT is most often a polymicrobial infection [123]. According to Kühn et al. [5], Streptococcus and Staphylococcus species are the most frequently isolated pathogens, often associated with upper respiratory tract infections. In our study, Pseudomonas aeruginosa was cultured in 6% of cases.

### 5.7. Intracranial Involvement

In cases with intracranial involvement, patients may present with signs of elevated intracranial pressure, including focal neurological deficits, nausea, and, in severe cases, loss of consciousness [124]. Our findings support this clinical picture: nausea and vomiting were reported in 30% of patients with intracranial involvement, while no such symptoms were observed in patients without it. Furthermore, altered mental status was present in 51% of patients with intracranial complications, compared to only 11.4% in those without, likely reflecting the mass effect exerted on the frontal lobes. In contrast, symptoms such as nasal congestion and the presence of a sinocutaneous fistula were more frequently observed in patients without intracranial extension.

### 5.8. Treatment Method

The cornerstone of PPT management is surgical drainage of the abscess. The choice of procedure depends on the extent of infection and individual anatomical considerations. In cases involving intracranial extension, external drainage—such as craniotomy—offers superior access and control, allowing for complete visualization of the frontal sinus and, when necessary, craniectomy for debridement of the infected bone [6]. Endoscopic sinus surgery (ESS), being less invasive, is associated with reduced morbidity and faster recovery. In our cohort, a combined surgical approach was most frequently utilized—typically involving initial external drainage followed by ESS, particularly in patients presenting with extensive swelling or osteomyelitis. Although some studies have reported an increasing trend toward the use of endoscopic techniques in recent years, especially for selected cases, current evidence does not conclusively establish ESS as the preferred modality in pediatric populations [1].

### 5.9. Antibiotic Therapy and Follow-Up

Antibiotic therapy is a cornerstone in the management of Pott’s puffy tumor (PPT) and should be initiated promptly after obtaining microbiological cultures. In our analysis, the mean total duration of antibiotic treatment was 61.9 days, comprising an average of 50.8 days of intravenous therapy and 33.2 days of oral therapy. Inappropriate empirical antibiotic selection was associated with prolonged treatment durations and the need for multiple adjustments to the therapeutic regimen. Given the average hospitalization period of 23 days, a well-structured outpatient follow-up system is crucial to ensure continuity of care. Notably, 80% of patients required rehospitalization; however, specific causes for readmission were rarely detailed in the literature, underscoring an area that warrants further investigation. The average follow-up period across studies was 14 months, during which three deaths were reported as a result of complications. Information regarding antibiotic selection was often limited. Most studies did not clearly distinguish between empirical and targeted therapy. Cephalosporins were the most frequently used first-line agents, likely due to their broad-spectrum activity. In some cases, antifungal agents were also administered. However, the interpretation of efficacy is limited by heterogeneity in treatment regimens, as cephalosporins were used both as monotherapy and in combination therapy. Importantly, antibiotic therapy alone is insufficient and is associated with a high rate of recurrence. Surgical intervention remains a critical component of definitive treatment [125].

## 6. Conclusions

Although PPT is a rare condition, it presents a significant clinical challenge. While antibiotic therapy remains a cornerstone of treatment, timely initiation of empirical therapy is critical and should occur after obtaining microbiological samples when possible. However, empirical treatment should not be delayed in situations where sample collection is not feasible, particularly in cases requiring urgent intervention or referral to tertiary care centers. Empiric therapy should be followed by targeted treatment, with consideration of antifungal agents when clinically indicated. The sudden appearance of a forehead mass, often associated with a frontal bone abscess, should raise suspicion for PPT—not only in pediatric patients but also in adults, who were the primary focus of our study. Optimal management requires a multidisciplinary approach involving otolaryngologists, emergency physicians, neurologists, and neurosurgeons. Further research is needed to improve care coordination and to define evidence-based best practices for both treatment and follow-up. Although PPT has traditionally been associated with pediatric populations, our findings highlight its growing clinical relevance among adults. Future investigations should aim to elucidate its pathophysiology, refine diagnostic strategies, and assess long-term outcomes in adult patients.

## 7. Limitations

Our study faced several limitations. We lacked data on intensive care unit stays, the duration of anesthesia, and blood transfusions, which limited the scope of our clinical analysis. Many publications did not provide detailed information on antibiotic regimens, such as duration, administration route, or specific drug combinations, making it difficult to assess treatment protocols. Additionally, surgical procedures were often inconsistently described, prompting us to categorize them into three general groups: ESS, a combined approach, and external drainage. Due to the variability in reporting, further stratification or analysis of surgical outcomes was not feasible.

## Data Availability

No new data were created or analyzed in this study.

## Supplementary Materials

The following supporting information can be downloaded at https://www.mdpi.com/article/10.3390/jcm14124062/s1, Table S1: Database.
