# Supplementary material for: Pott’s Puffy Tumor in the Adult Population: Systematic Review and Meta-Analysis of Case Reports
Source: J Clin Med. 2025 Jun 8;14(12):4062. doi: 10.3390/jcm14124062 (PMC12194702; doi:10.3390/jcm14124062)
Supplement: Supplementary file 1 [file jcm-14-04062-s001.zip › jcm-3627218-supplementary.pdf]

Year	1. Autor	Adult/Child	Age	Sex (F, M)	ck, asian, native a	intracranial involvement 0-NO, 1-YES	Cultured organisms	Antibiotic therapy	duration of oral antibiotic therapy,	duration of intravenous therapy	If yes, duration of antibiotic therapy (Days)	Surgical approach 0-no, 1-yes	Surgical approach	Dni hospitalizacji calkowitej	Follow up (months)
2012	Kosuke Akiyama et al.	Adult	37	M	Unknow	0	Not specified	Undefined	21	14	42	1	External drainage	None	None
2009	S. Collet et al.	Adult	54	M	White	0	Sterile	Amoxicillin	None	None	33	1	External drainage (Cairn Unterherger procedure)	18	None
2009	S. Collet et al.	Adult	76	M	Unknow	0	Streptococcus species	None	21	14	42	1	Combined approach, (External drainage, ESS)	None	None
2009	S. Collet et al.	Adult	38	M	Unknow	0	Prevotella oralis + Fusobacterium + Micromonas micros	None	21	21	42	1	Combined approach, (External drainage, ESS)	None	None
2009	S. Collet et al.	Adult	28	M	Unknow	0	Sterile	None	21	21	42	1	External drainage (External Frontal Ethmoidotomy)	None	None
1991	Gary A. Noskin et al.	Adult	34	M	Unknow	0	Bacteroides melaninogenicus, Eubacterium lentum, Fusobac-terium, Propionibacterium, and streptococcus species.	Ampicillin, Sulbactam, Amoxicillin with Clavulanic Acid	28	14	42	1	External drainage (External Frontal Sinus Surgery)	42	1
2007	Gareth Blackshaw et al.	Adult	54	F	Unknow	0	Sterile	Amoxicillin with Clavulanic Acid, Flucloxacillin, Penicillin	30	None	30	1	External drainage (External fronto-ethmoidectomy, Caldwell-Luc operation with antrostomy)	None	None
1996	Annelies Verbon et al.	Adult	83	M	Unknow	0	Haemophilus influenzae	None	28	14	42	1	External drainage (Revision of the frontal bone and surgical obliteration of the ethmoidal sinus)	None	None
1996	Annelies Verbon et al.	Adult	26	M	Unknow	0	Haemophilus influenzae	None	None	None	35	None	External drainage (External fronto-ethmoidectomy)	None	None
1997	G.J. Bellaney et al.	Adult	62	F	Unknow	0	Staphyloccocus species	Flucloxacillin	28		28	1	External drainage (Left-sided external fronto-ethmoidectomy)	None	None
1999	Paresh Shah et al.	Adult	78	F	White	0	Not specified	None	None	None	None	None	No surgical approach	None	None
2009	Binoy Chandy MD et al.	Adult	21	M	Unknow	1	Streptococcus species and Bacteroides melaninogeni-cus	Vancomycin, Metronidazole, Cephalosporin		28	28	1	External drainage (Bicoronal approach)	28	None
2002	Rachel Tattersall et al.	Adult	53	M	Unknow	1	Streptococcus species	Undefined	None	None	None	1	External drainage (Bifrontal craniotomy)	None	None
2002	S W Kung et al.	Adult	39	M	Unknow	1	Streptococcus species	Penicillin, Amoxicillin	21	21	42	1	External drainage (Drainage and debridement of right frontal sinus)	21	0,5
2003	Canbaz, B. et al.	Adult	67	F	Unknow	1	Pseudomonas aeruginosa	None	None	None	None	1	External drainage	None	0,67
2004	Goldfarb, A. et al.	Adult	58	M	Unknow	0	Not specified	Cephalosporin		56	56	1	External drainage (Osteoplastic surgery)	56	None
2005	Ç. Evliyaoglu et al.	Adult	27	M	Unknow	1	Staphylococcus species	Ampicillin, Sulbactam, Amoxicillin with Clavulanic Acid	42	42	84	1	External drainage	42	9
2005	Effat, K. G. et al.	Adult	62	F	African	0	Mucormycosis	Amphotericin, Efoperazone, Gentamicin, Metronidazole	None	None	None	1	External drainage	21	None
2007	N. Kaabia et al.	Adult	25	M	Unknow	1	Streile	Cephalosporin, Metronidazole, Gentamicin	64	10	64	1	Combined approach (Aspiration of right frontal and maxillary sinuses and incision of the supraorbital abscess)		12
2007	Raja V	Adult	49	M	White	0	Staphylococcus species	Amoxicillin with Clavulanate Acid, Flucloxacillin, Fusidic Acid, Metronidazole	7	42	49	1	Combined approach (Trephination of the right frontal sinus, Endoscopic Sinus Surgery)	49	None
2008	Minutilli, E. et al.	Adult	55	F	White	0	Staphylococcus species	Undefined	84		84	1	External drainage (Surgical drainage, debridement, and reconstruction of the bone)	None	24
2008	Lamoreau, K. P. et al.	Adult	55	M	White	0	Streptococcus species	Cephalosporin, Clindamycin, Vancomycin	None	None	0	0	No surgical approach	The patient left hospital against medical advice=	3
2008	Gabriel J. Martinez-Diaz et al.	Adult	54	F	African	0	Staphylococcus species + Streptococcus species	Penicillin	None	5	9	1	External drainage (Surgical drainage and reconstruction of the bone)	5	None
2011	Pamela D. Bannon et al.	Adult	41	M	Unknow	1	Streptococcus species	Cephalosporin, Metronidazole	None	None	None	1	Combined approach (Left frontal ethmoidectomy, Drainage of abscess)	20	None
2009	Masterson, L. et al.	Adult	56	M	White	0	Not specified	Undefined	None	None	28	1	Combined approach (Frontal sinus surgery using a combined endonasal and percutaneous approach)	None	3
2010	Sumit Upadhyay	Adult	29	M	Unknow	0	Staphylococcus species	Quinolone	None	None	None	1	Combined approach	None	None
2016	Diane Apostolakos, MD, MS et al.	Adult	20	M	Unknow	1	Not specified	Ampicillin, Sulbactam, Metronidazole, Cephalosporin	90	51	90	0	No surgical approach	39	None
2019	Ibtisam Hasan et al.	Adult	75	F	Unknow	1	Streptococcus species	Doxycycline, Amoxicillin with Clavulanic Acid, Cephalosporin	14	45	59	1	Combined approach (Surgical drainage, frontal sinus trephination and endoscopic frontal sinusotomy)	None	None
2012	Jinho Jung, MD et al.	Adult	25	M	Asian	0	Staphylococcus species	Cephalosporin	9	56	65	1	ESS (Endoscopic ethmoidectomy, drainage of the abscess)	9	None
2019	Petros Koltsidopoulos et al.	Adult	37	F	White	1	Staphylococcus species	Undefined		42	42	1	Combined approach (Combined endoscopic and external surgical approach)	None	None
2017	Aleksandar Perić et al.	Adult	33	M	Unknow	1	Staphylococcus species, anaerobe Peptostreptococcus prevoti, Streptococcus species, Haemophilus influenzae,Staphylococcus species	Cephalosporin, Metronidazole, Amoxicillin with Clavulanic Acid	28	28	56	1	External drainage (Craniotomy; bilateral endoscopic anterior ethmoidectomy)	28	84
2013	Ayub ur Rehman et al.	Adult	27	M	White	0	Not specified	Undefined		70	70	0	No surgical approach	None	None
2015	N.A. van der Poel et al.	Adult	23	M	Unknow	0	Staphylococcus species, Streptococcus species	Clindamycin	None	42	42	1	ESS (Draf IIa)	None	None
2014	Rajwani K. M. et al.	Adult	61	M	Unknow	0	Not specified	Undefined	None	None		1	Combined approach (Surgical drainage, ESS)	None	recurrent PPT
2015	Hitoshi Terui, MD et al.	Adult	52	F	Asian	0	Not specified	Undefined	None	None	None	1	Combined approach (Endoscopic Sinus Surgery, Killian frontal sinus surgery, reconstruction of the right nasofrontal duct)	None	
2019	Makarowicz, M. et al.	Adult	73	F	White	0	Not specified	Amoxicillin with Clavulanic Acid, Azithromycin, Clindamycin, Cephalosporin	None	None	unspecifed	1	ESS (FESS)	unspecified	None
2019	Makarowicz, M. et al.	Adult	47	M	White	0	Not specified	Quinolone	10		10	1	Combined approach (Surgical drainage, FESS)	unspecified	None
2016	Satomi Tatsumi et al.	Adult	46	M	Asian	0	Sterile	Cephalosporin	None	None	None	1	Combined approach (Surgical drainage, ESS)	Unknown	None
2022	Kühn, J.P. et al.	Adult	19	M	Unknow	0	Streptoccocus species	Cephalosporin	None	9	9	1	ESS (FESS)	9	None
2011	Ketenci et al.	Adult	41	F	Unknow	1	Streptococcus species	Ampicillin, Sulbactam, Vancomycin, Meronem, Netilmycin	None	None	None	1	Combined approach (External drainage, ESS, Craniotomy, Craniectomy)	None	0
2011	Ketenci et al.	Adult	60	M	Unknow	0	Proteus spp,	Ampicillin, Sulbactam	None	None	None	1	Combined approach (External frontal approach, bilateral ESS)	None	6
2011	Ketenci et al.	Adult	27	M	Unknow	0	AnerobicGram (+) diplococci	Ampicillin, Sulbactam, Vancomycin, Meronem, Netilmycin	None	None	None	1	Combined approach (External drainage, ESS)	None	36
2011	Ketenci et al.	Adult	24	M	Unknow	1	Echerichia coli Staphyloccocus species	Ampicillin, Sulbactam, Meronem, Netilmycin	None	None	None	1	Combined approach (External drainage, ESS)	None	3
2022	Yang, Hui Joon MD et al.	Adult	48	M	Asian	0	Not specified	Cephalosporin, Amikacin, Clindamycin, Quinolone	None	None	60	1	Combined approach (FESS, frontal sinus trephination)	14	12
1998	Robert Skomro et al.	Adult	58	F	White	1	Pasteurella multocida	Cephalosporin, Clindamycin, Penicillin	40	35	40	1	Lynch procedure	7	
2020	Min HJ et al.	Adult	37	F	Asian	0	Not specified	None		21	21	1	ESS (Draf IIA)		6
2024	Shuya Tatsuki et al.	Adult	56	F	Asian	0	Not specified	Ampicillin, Sulbactam, Cephalosporin	16	19	35	1	ESS	19	4
2023	Kaltham Al Doaibel et al.	Adult	32	M	Saudi	1	Staphyloccocus species	Cephalosporin, Clindamycin, Vancomycin	28	14	42	1	External drainage (Craniotomy)	42	None
2024	Ananth P. Abraham et al.	Adult	32	F	Unknow	0	Mucormycosis	Amphotericin B, Posaconazole	14	166	180	1	Combined approach (Endoscopic clearance, Craniectomy, Lynch maneuver)	None	23
2024	Ananth P. Abraham et al.	Adult	55	M	Unknow	0	Mucormycosis	Amphotericin B, Posaconazole	27	144	171	1	Combined approach (Endoscopic clearance, Craniectomy, Lynch maneuver)	None	23
2024	Ananth P. Abraham et al.	Adult	61	F	Unknow	1	Mucormycosis	Amphotericin B, Posaconazole	28	155	178	1	Combined approach (Endoscopic clearance, Craniectomy, Lynch maneuver)	None	23
2024	Ananth P. Abraham et al.	Adult	59	M	Unknow	0	Mucormycosis	Amphotericin B, Posaconazole	14	135	150	1	Combined approach (Endoscopic clearance, Craniectomy, Lynch maneuver)	None	5
2024	Ananth P. Abraham et al.	Adult	54	M	Unknow	0	Mucormycosis	Amphotericin B, Posaconazole	10	345	355	1	Combined approach (Endoscopic clearance, Craniectomy, Lynch maneuver)	None	21
2024	Ananth P. Abraham et al.	Adult	46	M	Unknow	0	Mucormycosis	Amphotericin B, Posaconazole	14	186	200	1	Combined approach (Endoscopic clearance, Craniectomy, Lynch maneuver)	None	21
2024	Ananth P. Abraham et al.	Adult	48	M	Unknow	0	Mucormycosis	Amphotericin B, Posaconazole	14	346	360	1	Combined approach (Endoscopic clearance, Craniectomy, Lynch maneuver)	None	20
2024	Ananth P. Abraham et al.	Adult	65	M	Unknow	0	Mucormycosis	Amphotericin B, Posaconazole	28	420	448	1	Combined approach (Endoscopic clearance, Craniectomy, Lynch maneuver)	None	17
2024	Ananth P. Abraham et al.	Adult	46	M	Unknow	0	Mucomycosis - R. arthitus	Amphotericin B, Posaconazole	14	135	159	1	Combined approach (Endoscopic clearance, Craniectomy, Lynch maneuver)	None	19
2024	Kuylhee Kim et al.	Adult	76	F	Asian	1	Not specified	Undefined	None	None	None	1	Combined approach (Surgical excision of abscess ESS)	5	12
1984	L. M. Gil-Carcedo et al.	Adult	48	M	White	1	Steptococcus species	Undefined	60	None	None	1	Combined approach	None	10
1984	L. M. Gil-Carcedo et al.	Adult	39	M	White	1	Sterile	Undefined	None	None	None	1	External drainage (Cranioctomy)	None	None
1985	Koch, S. E. et al.	Adult	74	M	Unknow	1	Staphyloccocus species	Nafcillin, Dicloxacillin	38	30	68	1	External drainage (Drainage of the abscess)	None	None
1989	David W. Kennedy MD et al.	Adult	37	M	Unknow	0	Not specified	None	None	None	None	1	ESS	None	21
1989	David W. Kennedy MD et al.	Adult	76	M	Unknow	0	Not specified	None	None	None	None	1	ESS	None	None
1989	David W. Kennedy MD et al.	Adult	62	F	Unknow	0	Haemopihlus influeza, Proteus mirabilis, staphyloccous species	Undefined	None	None	None	1	ESS	None	5
1997	Banooni et al.	Adult	34	F	Unknow	1	Streptococcus species, candida albicans	Clindamycin, Cephalosporin, Quinolone, Metronidazole	42	42	42	1	Combined approach (ESS, surgical clearance)	14	None
1996	R P Babu et al.	Adult	20	M	Unknow	1	Streptococcus species	Penicillin	None	42	42	1	External drainage (A bifrontal craniotomy via a standard bicoronal skin incision)	42	None
2001	Nicholas C. Bambakidis et al.	Adult	18	M	Unknow	1	Microaerophilic, streptococcus	Cephalosporin, Metronidazole		42	42	1	External drainage (Bifrontal craniotomy)	42	12
2001	Bağdatoğlu C. et al.	Adult	18	M	Unknow	1	Gram positive cocci	Vancomycin, Rifampicin, Metronidazole	56	61	61	1	Combined approach (Craniectomy, intranasal surgery)	90	12
2001	Karabatsou K. et al.	Adult	29	M	Unknow	1	Sterile	Flucloxacillin	None	None	None	1	External drainage (Craniotomy, surgical drainage and reconstruction of the bone)	None	None
2002	Lang E. E. et al.	Adult	44	M	Unknow	1	Sterile	Cephalosporin, Metronidazole	None	None	None	1	External drainage (Frontal trephine, antral washout, craniotomy)	None	None
2002	Lang E. E. et al.	Adult	21	M	Unknow	1	Sterile	Cephalosporin, Metronidazole	None	None	None	1	External drainage (Frontal trephine, antral washout, craniotomy)	None	None
2002	Lang E. E. et al.	Adult	23	M	Unknow	1	Streptoccocus species	Cephalosporin, Metronidazole	None	None	None	1	External drainage (Frontal trephine, antral washout, craniotomy)	None	None
2002	Lang E. E. et al.	Adult	58	M	Unknow	1	Sterile	Cephalosporin, Metronidazole	None	None	None	1	External drainage (External fronto-ethmoidectomy, craniotomy)	None	4
2003	Chow K. M. et al.	Adult	60	F	Asian	0	Mycobacterium tuberculosis.	None	None	None	None	None	No surgical approach	None	
2004	Rakesh K. Chandra et al.	Adult	42	F	Unknow	0	Not specified	None	None	None	None	1	ESS (Draf IIB)	None	6
2004	Chaturvedil V. N. et al.	Adult	50	F	Unknow	1	Sterile	Undefined	None	None	180	1	External drainage (Lynch, Howarth recision)	7	1
2005	Adame N. et al.	Adult	21	M	White	1	Staphylococcus aureus, Escherichia coli	Undefined	None	None	None	1	ESS	None	None
2005	Naresh Kumar Panda et al.	Adult	47	F	Unknow	0	Aspergillus flavus	None	None	None	None	1	ESS (Frontoethmoidectomy and debridement, endoscopy clerance after 3 months)	None	14
2005	Naresh Kumar Panda et al.	Adult	35	M	Unknow	0	Aspergillus flavus	None	None	None	None	1	External drainage	None	3
2007	McDermott C. et al.	Adult	21	M	White	0	G+ cocci	Penicillin, Metronidazole	14	28	42	1	External drainage (Trephination and drainage)	28	
2008	Lyndal J Tacon et al.	Adult	62	F	Unknow	0	Prevotella sp	Clindamycin, Ertapenem, Metronidazole	42	42	84	1	External drainage	14	12
2010	Adejumo A. et al.	Adult	32	M	Unknow	1	Sterile	Ampicillin, Sulbactam, Amoxicillin with Clavulanic Acid	28	14	42	1	External drainage (Frontal craniotomy and evacuation of abscess)	14	2
2011	Oladejo Olaleye et al.	Adult	60	M	White	0	Not specified	Undefined	None	None	None	1	Combined approach (Endoscopic balloon sinuplasty sinus surgery)	None	None
2011	Bhatt, Y. M. et al.	Adult	60	F	White	1	Not specified	Cephalosporin, Metronidazole, Vancomycin		42	42	1	Combined approach (Endoscopic sinus drainage with external frontal sinus trephine)	None	None
2011	Sajid T. et al.	Adult	51	M	Unknow	0	Not specified	Undefined	None	None	None	1	External drainage	None	None
2012	Elyassi, A. R. et al.	Adult	21	M	Unknow	1	Eikenella corrodens, Prevotella bivia and Streptococcus species	Cephalosporin, Vancomycin, Metronidazole, Quinolone	7	24	31	1	External drainage (Caldwell-Luc procedure, craniotomy)	30	6
2012	Harpreet Singh Grewal et al.	Adult	38	M	Unknow	0	Eubacterium species and Staphylococcus species	Vancomycin, Cephalosporin, Metronidazole, Clindamycin	3	42	45	1	ESS	42	None
2012	Ellen Welch et al.	Adult	52	M	Unknow	0	non metioned	Cephalosporin	None	None	None	1	ESS (FSS)	None	None
2012	Özcan H.N. et al.	Adult	27	M	Unknow	0	Not specified	Undefined	None	None	None	1	ESS	None	None
2012	Kazım Bozdemir et al.	Adult	52	M	Unknow	0	Sterile	Cephalosporin, Metronidazole, Quinolone	42	24	66	1	Balloon procedure (Balloon catheter dilatation)	24	10
2012	Kazım Bozdemir et al.	Adult	41	M	Unknow	0	Sterile	Cephalosporin, Metronidazole, Quinolone	42	35	77	1	Balloon procedure (Balloon catheter dilatation)	35	11
2012	Kazım Bozdemir et al.	Adult	50	F	Unknow	0	Sterile	Cephalosporin, Metronidazole, Quinolone	42	28	70	1	Balloon procedure (Balloon catheter dilatation)	28	11
2012	Shin, J. W. et al.	Adult	72	F	Asian	1	Staphylococcus species	Cephalosporin		7	7	1	Combined approach (Excision and curettage through combined approach)	7	12
2012	Domville-Lewis, C. et al.	Adult	21	F	Native australian	1	Streptococcus species	Meropenem, Vancomycin, Cephalosporin, Metronidazole, Amoxicillin	28	32	60	1	Combined approach (Percutaneous drainage, ESS)	11	21
2013	Sullivan C. D. et al.	Adult	61	M	African	0	Not specified	Amoxicillin with Clavulanic Acid	None	None	None	1	Combined approach (Sinustrephination, ESS)	None	8
2014	Adela Magdalena Ciobanu et al.	Adult	37	M	Unknow	1	Mycoplasma	Doxycycline	None	None	None	1	Unknown	None	2
2014	Stephen P Williams et al.	Adult	63	F	White	0	Streptococcus species	Amoxicillin with Clavulanic Acid	None	None	None	1	Combined approach (External drainage of the abscess and ESS)	None	None
2015	Stephen L Ball et al.	Adult	-	M	White	0	Streptococcus species	Metronidazole, Quinolone	None	None	90	1	Combined approach (ESS and frontal sinus trephines)	None	None
2015	Sang Hun Hyun et al.	Adult	55	M	Unknow	0	cus species, Pseudomonas aeruginosa, Enterococcus faecium	Cephalosporin, Quinolone, Voriconazole	None	None	28	1	External drainage (Surgical drainage and debridement with combined bilateral frontal bone craniectomy)	28	
2016	Clarós, P. et al.	Adult	37	M	White	0	Sterile	Quinolone	None	28	28	1	External drainage (Bicoronal incision)	None	6
2016	Brandon Miller et al.	Adult	60	M	African	1	Not specified	Vancomycin, Cephalosporin, Metronidazole	None		42		Unknown	None	
2017	Satvinder Singh Bakshi et al.	Adult	36	M	Unknow	0	Not specified	Undefined	None	42	42	1	ESS	42	6
2022	Ripu Daman Arora et al.	Adult	33	M	Native	1	Mucormycosis	Undefined	None	None	None	None	Combined approach (Endoscopic procedure with bicoronal incision frontal craniectomy)	None	1
2022	Salemans R. et al.	Adult	84	M	White	0	Not specified	Clindamycin, Amoxicillin with Clavulanic Acid	49	None	49	1	ESS	9	None
2022	Hitt N. et al.	Adult	76	F	Unknow	0	Not specified	Amoxicillin with Clavulanic Acid	42	None	42	0	No surgical approach	None	None
2022	Mahmoud S. Abbas et al.	Adult	37	M	Unknow	0	Staphylococcus species	Cephalosporin, Metronidazole, Vancomycin, Nafcillin	42	None	42	1	External drainage (External sinus surgery and craniectomy)	6	None
2023	Eric W. Lai et al.	Adult	33	M	African	0	Not specified	Undefined	None	None	None	1	External drainage	3	None
2017	Şimşek, H. et al.	Adult	65	M	White	1	Pseudomonas aeruoginosa	Cephalosporin, Metronidazole, Quinolone	None	42	42	1	External drainage	None	3
2018	Sekine R. et al.	Adult	56	M	Asian	1	Stenotrophomonas maltophilia, Achromobacter xylosoxidans, and Corynebacterium spp	Vancomycin, ABPC, SBT, Quinolone	None	56	56	1	Combined approach (Surgical drainage + Draf III)	None	15
2019	Keli Dusu et al.	Adult	35	M	Unknow	1	Not specified	Cephalosporin	None	None	28	1	ach (Emergency endoscopic drainage of the sinuses and drainage of the frontal abscess via an external left e	28	None
2019	Hyun Jin Min et al.	Adult	49	M	Unknow	0	Not specified	Undefined	28	None	28	1	ESS	None	None
2020	Alfonso Luca Pendolino et al.	Adult	26	M	Unknow	0	Not specified	Clarithromycin	None	None	56	1	ESS (Draf III)	14	13
2020	Alfonso Luca Pendolino et al.	Adult	59	M	Unknow	0	Not specified	Clarithromycin	None	None	56	1	ESS (Draf III)	14	9
2020	Alfonso Luca Pendolino et al.	Adult	51	M	Unknow	1	Not specified	Clarithromycin	None	None	56	1	ESS (Draf III)	14	18
2020	Alfonso Luca Pendolino et al.	Adult	62	M	Unknow	0	Streptococcus species	Clarithromycin	None	None	56	1	Combined approach (ESS + external drainage + Draf III)	14	5
2020	Ekta Paw et al.	Adult	34	M	Unknow	1	G+ bacilii	Amoxicillin with Clavulanic Acid	None	None	None	1	Combined approach (ESS, maxillary antrostomy and sphenoidectomy)	None	12
2020	Holly Bean et al.	Adult	53	F	White	0	Actinomyces naeslundi.	Cephalosporin, Doxycycline	117	63	180	1	Combined approach (ESS, surgical debridement of the abscess with frontal sinus trephination)	None	None
2020	Michael W Song et al.	Adult	31	M	Unknow	1	Not specified	Vancomycin, Cephalosporin, Metronidazole, Quinolone, Linezolid	42	5	47	1	Combined approach (bilateral sinusotomy, total ethmoidectomy, sphenoidotomy, maxillary antrostomy, and inferior turbinate reduction.)	5 (against medical advice)	1
2020	Sideris G. et al.	Adult	50	M	Unknow	1	Staphylococcus species	Undefined	10	46	56	1	Combined approach (ESS, surgical drainage of mucocele)	13	None
2020	Sideris G. et al.	Adult	47	M	Unknow	0	Staphylococcus species	Undefined	33	10	43	1	External drainage(Sinus treparation)	10	None
2020	Pansini, A. et al.	Adult	45	F	White	0	Staphylococcus species	Amoxicillin with Clavulanic Acid	14	None	14	1	Combined approach (FESS with medium bilateral antrostomy, anteroposterior bilateral ethmoidectomy and bilateral sphenoidectomy)	None	None
2020	Marina Yamamotoa et al.	Adult	45	M	Unknow	0	Sterile	Cephalosporin, Clindamycin, Fosfomycin	None	None	None	1	ESS (Draf III)	None	None
2021	Birks S. et al.	Adult	27	F	Unknow	1	Not specified	Undefined	None	None	None	1	Unknown	None	None
2021	Baker N. et al.	Adult	40	M	African	0	Streptococcus species	Amoxicillin with Clavulanic Acid	None	42	42	1	Combined approach (Anterior orbitotomy with abscess drainage + ESS)	None	None
2020	Thompson, H. M. et al.	Adult	46	M	Unknow	1	Staphylococcus species	Undefinied	None	42	42	1	External drainage (Cranialization + FTT)	None	90
2020	Thompson, H. M. et al.	Adult	18	F	Unknow	0	Staphylococcus species	Undefined	None	42	42	1	ESS (Draf III)	None	8

2020	Thompson, H. M. et al.	Adult	60	M	Unknow	0	staphyloccocus species	Undefined	None	42	42	1	ESS (Reidel)	None	10
2020	Thompson, H. M. et al.	Adult	38	F	Unknow	0	Pseudomonas aeruginosa	Undefined	None	42	42	1	External drainage (OPF + FTT)	None	24
2020	Thompson, H. M. et al.	Adult	52	M	Unknow	0	staphyloccocus species	Undefined	None	42	42	1	Combined approach (Draf III + Lynch)	None	16
2020	Thompson, H. M. et al.	Adult	84	M	Unknow	0	eudomonas aeruginosa, Proteus mirablis, Klebsiella pneumoni	Undefined	None	42	42	1	ESS (Draf III)	None	18
2020	Thompson, H. M. et al.	Adult	30	F	Unknow	0	Staphyloccocus species	Undefined	None	42	42	1	Combined approach (Draf III + OPF)	None	8
2020	Thompson, H. M. et al.	Adult	48	M	Unknow	0	staphyloccocus species	Undefined	None	42	42	1	ESS (Draf IIB)	None	11
2020	Thompson, H. M. et al.	Adult	22	F	Unknow	0	Sterile	Quinolone	None	42	42	1	External drainage (OPF + FO)	None	11
2020	Thompson, H. M. et al.	Adult	74	M	Unknow	0	Sterile	Quinolone	None	42	42	1	ESS (Reidel)	None	12
2020	Thompson, H. M. et al.	Adult	52	F	Unknow	0	Pseudomonas aeruginosa	Undefined	None	42	42	1	Combined approach (Reidel, removal of plates)	None	6
2020	Thompson, H. M. et al.	Adult	69	M	Unknow	0	Pseudomonas aeruginosa	Undefined	None	42	42	1	Combined approach (Draf IIB, Excision of fistula)	None	72
2020	Thompson, H. M. et al.	Adult	52	M	Unknow	0	staphyloccocus species	Undefined	None	42	42	1	ESS (Draf IIB)	None	24
2020	Thompson, H. M. et al.	Adult	65	M	Unknow	0	Pseudomonas aeruginosa	Undefined	None	42	42	1	ESS (Draf III)	None	24
2020	Thompson, H. M. et al.	Adult	54	F	Unknow	0	Pseudomonas aeruginosa	Undefined	None	42	42	1	ESS (Draf III)	None	50
2021	Josie Acuña et al.	Adult	59	M	Unknow	0	Not specified	None	None			1	ESS	None	None
2020	Hyun Jin Min et al.	Adult	37	M	Asian	0	Sterile	Undefined	None	21	21	1	ESS	None	6
2017	Low et al.	Adult	79	F	White	0	Streptococcus species	Undefined	None	None		1	ESS	None	None
2017	Leong, S. C. et al.	Adult	57	M	Unknow	0	Sterile	None	42	None	42	1	ESS (Draf III)	None	None
2017	Leong, S. C. et al.	Adult	29	M	Unknow	0	Sterile	None	42	None	42	1	ESS (Draf III)	None	None
2017	Leong, S. C. et al.	Adult	86	F	White	0	Staphylococcus species, Pseudomonas, Bacteroides	None	None	42	42	1	Balloon procedure (Balloon sinuplasty)	None	None
2017	Preethi Ganapathy et al.	Adult	77	F	White	0	Not specified	Undefined	None		-	1	ESS	None	None
2018	Federico Pasin et.al.	Adult	81	M	White	0	Streptococcus species	None	None	42	42	1	ESS	None	None
2017	Thomas Geyton et al.	Adult	45	M	White	0	Not specified	None	None			1	ESS	None	None
2018	Michael S. Morris et al.	Adult	55	F	White	0	Sterile	, Sulbactam, Cephalosporin, Amoxicillin with Clavulanic Acid, Er	7	42	49	1	Combined approach (Endoscopic left frontal and maxillary sinus operation with balloon sinuplasty, left anterior ethmoidectomy with frontal stent placement)	None	None
2018	Alexandre Simonin et al.	Adult	21	M	White	1	Streptococcus species	Cephalosporin	None	56	56	1	External drainage (A bicoronal skin incision)	None	None
2018	Saloni P. et al.	Adult	no access	Unknown	Unknow	0	Not specified	None	None	None	None	None	No surgical approach	None	None
2019	B Mettias et al.	Adult	55	M	Asian	0	streptococcus species	Clarithromycin, Amoxicillin with Clavulanic Acid, Doxycycline, Budesonide, Metronidazole, Cephalosporin, Penicillin	63	28	63	1	External drainage (Craniectomy with debridement and distraction osteogenesis)	None	None
2022	Kapil Mani KC et al.	Adult	63	M	Asian	0	Sterile	Cephalosporin, Clindamycin	83	7	90	1	External drainage (Decompression and debridement of the wound, drainage of sinus)	None	None
2020	Carmen Salom-Covenas et al.	Adult	61	M	White	0	Not specified	Vancomycin		7	7	1	Combined approach (Draf IIA, surgical drainage and trephination, further Draf III)	7	None
1980	Daniel Remmler et al.	Adult	25	M	Unknow	1	streptococcus species	Penicillin	21	14	35	1	External drainage (Left frontal sinusotomy)	14	None
1980	Daniel Remmler et al.	Adult	20	M	Unknow	1	Streptococcus species	Tetracycline, Nafcillin, Chloramphenicol, Penicillin	21	7	28	1	External drainage (Frontal sinus trephination, craniotomy)	7	None
1980	Daniel Remmler et al.	Adult	22	M	Unknow	1	Streptococcus species	Penicillin	21	14	35	1	External drainage (Craniotomy)	14	None
1998	Richard M. Gallagher et al.	Adult	43	F	Unknow	1	Sterile	Cephalosporin, Nafcillin, Metronidazole	None	36	36	1	Unknown	None	None
1998	Richard M. Gallagher et al.	Adult	28	M	Unknow	1	Sterile	Cephalosporin, Nafcillin, Metronidazole	None	36	36	1	Unknown	None	None
1998	Richard M. Gallagher et al.	Adult	48	M	Unknow	1	Sterile	Cephalosporin, Nafcillin, Metronidazole	None	36	36	1	Unknown	None	None
1998	Richard M. Gallagher et al.	Adult	36	M	Unknow	1	streptococcus species	Cephalosporin, Nafcillin, Metronidazole	None	36	36	1	Unknown	None	None
1998	Richard M. Gallagher et al.	Adult	19	M	Unknow	1	crophorum, Peptostreptococcus asaccharolyticus, Bacteriodes	Cephalosporin, Nafcillin, Metronidazole	None	36	36	1	Unknown	None	None
1998	Richard M. Gallagher et al.	Adult	24	F	Unknow	1	Fusobacterium nucleatum	Cephalosporin, Nafcillin, Metronidazole	None	36	36	1	ESS (Two external ethmoidectomies)	None	None
1998	Richard M. Gallagher et al.	Adult	52	M	Unknow	1	Sterile	Cephalosporin, Nafcillin, Metronidazole	None	36	36	1	ESS	None	None
1998	Richard M. Gallagher et al.	Adult	56	F	Unknow	1	Streptococcus species	Cephalosporin, Nafcillin, Metronidazole	None	36	36	1	Unknown	None	None
1998	Richard M. Gallagher et al.	Adult	28	M	Unknow	1	Sterile	Cephalosporin, Nafcillin, Metronidazole	None	36	36	1	Unknown	None	None
1998	Richard M. Gallagher et al.	Adult	51	F	Unknow	1	Streptococcus species	Cephalosporin, Nafcillin, Metronidazole	None	36	36	1	Combined approach (Osteoplastic flap and frontal sinus obliteration)	None	None
1998	Richard M. Gallagher et al.	Adult	65	M	Unknow	1	Sterile	Cephalosporin, Nafcillin, Metronidazole	None	36	36	1	Unknown	None	None
2018	Juan Casado Pellejero et al.	Adult	52	M	Unknow	1	pionibacterium acnes, Actinomyces sp y Fusobacterium nuclea	Vancomycin, Cephalosporin, Metronidazole, Amoxicillin	120	28	148	1	External drainage (Craniotomy and drainage of the abscesses)	None	None
2011	Acke F et al.	Adult	24	M	Unknow		Not specified	Undefined	None	None	None	None	No surgical approach	None	None
2016	Apostolakos D. et al.	Adult	20	M	Unknow	1	Not specified	Ampicillin, Sulbactam, Cephalosporin, Metronidazole	None	92	92	0	No surgical approach	39	None
2023	Jalali E. et al.	Adult	28	M	Unknow	1	Not specified	Undefined	None	None	None	1	Unknown	None	None
2023	Jalali E. et al.	Adult	54	M	Unknow	1	Not specified	Undefined	None	None	None	1	Unknown	None	16
2024	Tatsuki S et al.	Adult	56	F	Asian	0	Not specified	Ampicillin, Sulbactam, Cephalosporin	16	19	35	1	Combined approach (Drainage of the abscess, bilateral endoscopic sinus surgery)	19	4
2011	Kougaku Umezawa et al.	Adult	46	M	Asian	1	Not specified	Undefined	None	None	42	1	External drainage (Drainage of the abscess and debridement)	None	None
2010	M N Stienen et al.	Adult	26	M	White	0	Streptococcus species	Cephalosporin, Rifampicin, Vancomycin, Metronidazole	None	42	42	1	ESS	42	4
2016	Stephen L Ball et al.	Adult	65	M	White	0	Not specified	None	None	None	56	None	No surgical approach	None	None
2018	S Y Zhang et al.	Adult	45	M	White	0	Klebsiella pneumonie	None	None	None	None	ouldn't get access	Unknown	14	None
1977	Thomas, J. N. et al.	Adult	19	M	Unknow		Not specified	Sulphadiazine, Chloramfenicol	None	42	42	1	External drainage (Craniectomy)	42	None
2022	Shivdas et al.	Adult	71	M	White	0	Sterile	Flucloxacillin	23	14	37	1	Combined approach (ESS and extrernal drainage)	14	None
2024	Our case	Adult	40	M	White	1	Pseudomonas aeruginosa	Cephalosporin, Sulbactam	-			1	External drainage (Craniotomy and drainage of the abscesses)	10	None
